# Supplementary figures and images for: OsCAF1, a CRM Domain Containing Protein, Influences Chloroplast Development
Source: Int J Mol Sci. 2019 Sep 6;20(18):4386. doi: 10.3390/ijms20184386 (PMC6770308; doi:10.3390/ijms20184386)

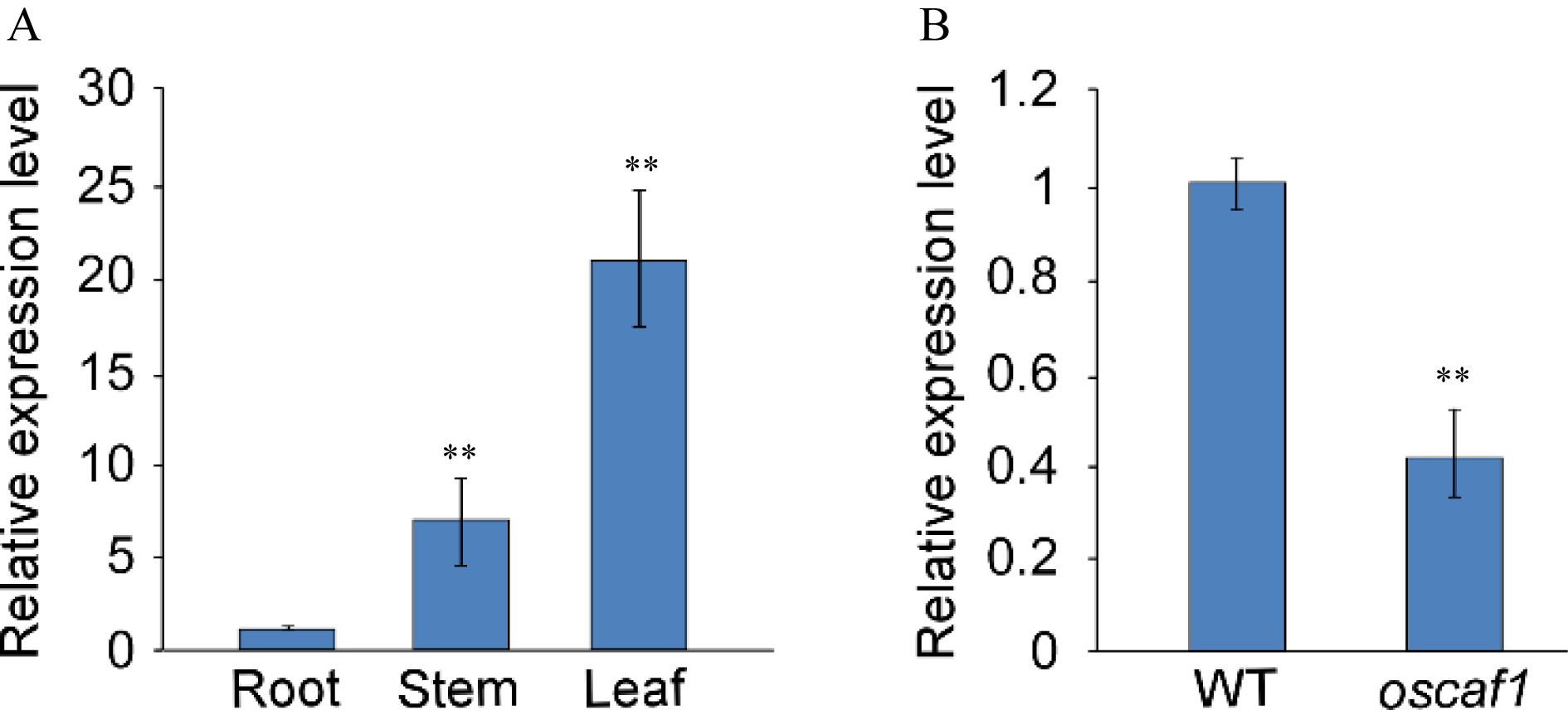

Supplement: Supplementary file 1 [file ijms-20-04386-s001.zip › ijms-556104-SI/Supplementary/Supplementary Figure S1 Analysis of OsCRS2 expression .tif]

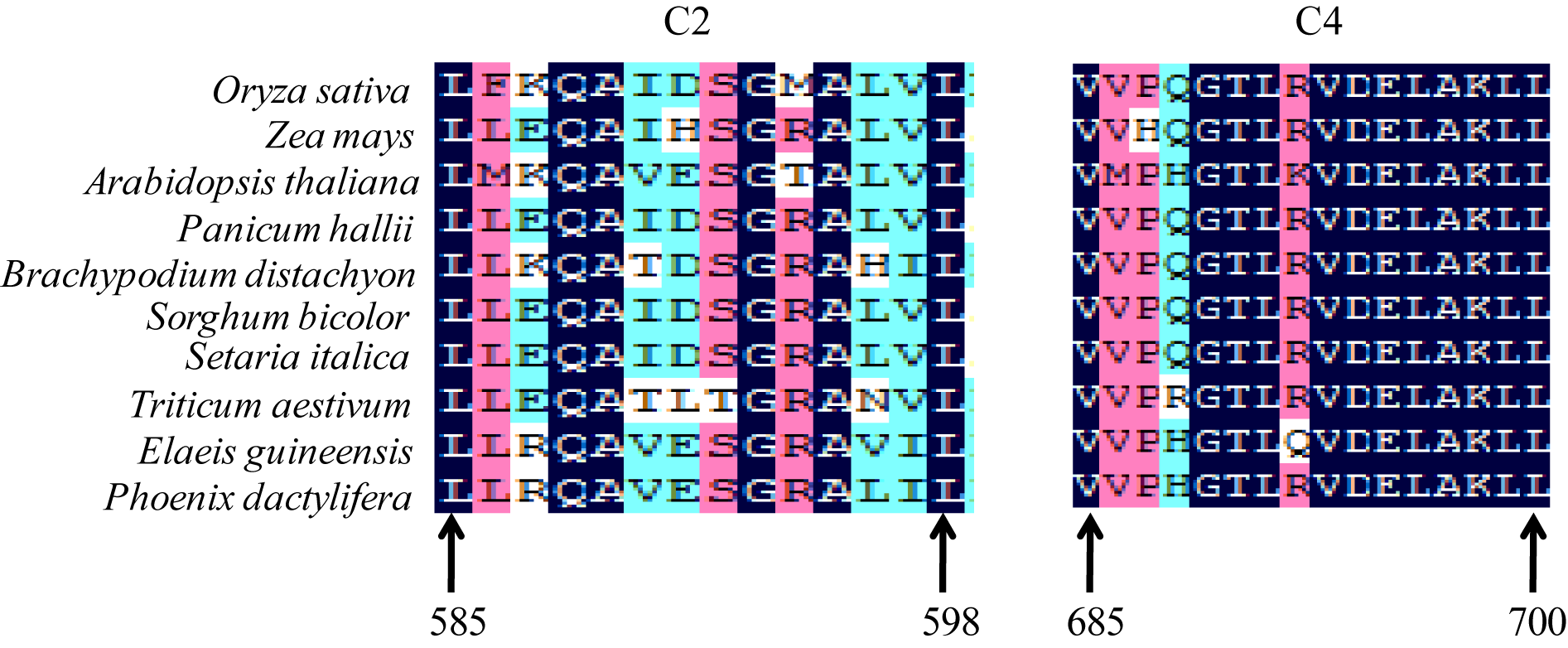

Supplement: Supplementary file 1 [file ijms-20-04386-s001.zip › ijms-556104-SI/Supplementary/Supplementary Figure S2 Comparative analysis of CAF1-C2 and C4 conserved sequences in various species.tif]

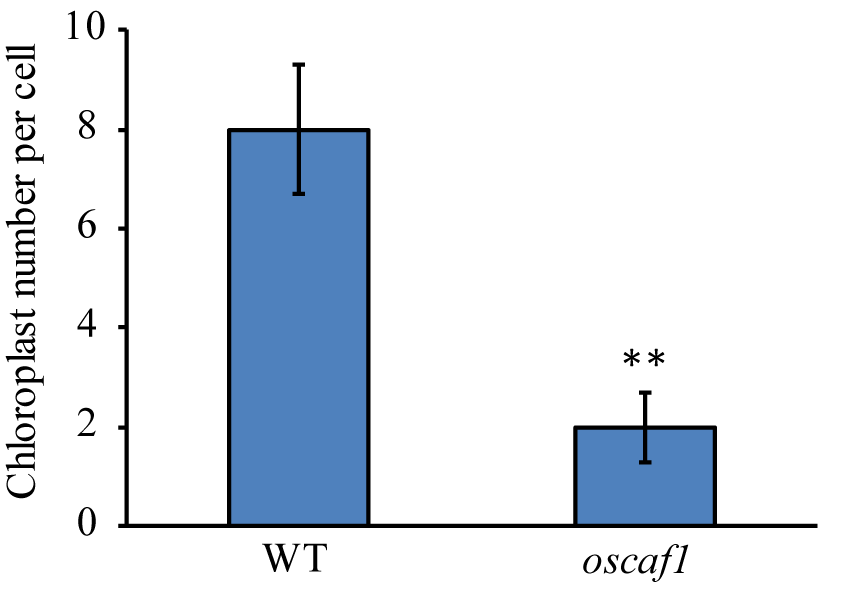

Supplement: Supplementary file 1 [file ijms-20-04386-s001.zip › ijms-556104-SI/Supplementary/Supplementary Figure S3 Comparison of chlorophyll number in mesophyll cells of wild type and mutant.tif]
